# Cortical alpha oscillations as a tool for auditory selective inhibition

**DOI:** 10.3389/fnhum.2014.00350

**Published:** 2014-05-28

**Authors:** Antje Strauß, Malte Wöstmann, Jonas Obleser

**Affiliations:** ^1^Max Planck Research Group “Auditory Cognition”, Max Planck Institute for Human Cognitive and Brain SciencesLeipzig, Germany; ^2^International Max Planck Research School on Neuroscience of Communication, Max Planck Institute for Human Cognitive and Brain SciencesLeipzig, Germany

**Keywords:** alpha, neural oscillations, effortful listening, inhibition, masking, speech, aging, hearing loss

## Abstract

Listening to speech is often demanding because of signal degradations and the presence of distracting sounds (i.e., “noise”). The question how the brain achieves the task of extracting only relevant information from the mixture of sounds reaching the ear (i.e., “cocktail party problem”) is still open. In analogy to recent findings in vision, we propose cortical alpha (~10 Hz) oscillations measurable using M/EEG as a pivotal mechanism to selectively inhibit the processing of noise to improve auditory selective attention to task-relevant signals. We review initial evidence of enhanced alpha activity in selective listening tasks, suggesting a significant role of alpha-modulated noise suppression in speech. We discuss the importance of dissociating between noise interference in the auditory periphery (i.e., energetic masking) and noise interference with more central cognitive aspects of speech processing (i.e., informational masking). Finally, we point out the adverse effects of age-related hearing loss and/or cognitive decline on auditory selective inhibition. With this perspective article, we set the stage for future studies on the inhibitory role of alpha oscillations for speech processing in challenging listening situations.

## 1. Introduction

In ecological listening situations, auditory signals are rarely perceived in quiet due to the presence of different auditory maskers such as distracting background speech or environmental noise. Thus, sounds from different sources greatly overlap spectro-temporally at the level of the listener's ear. What are the neural correlates that facilitate selective listening to relevant target signals despite irrelevant auditory input (i.e., the “cocktail party problem”; Cherry, 1953)? At the central neural level, two complementary mechanisms of top–down control (i.e., regulation of subsidiary cognitive processes) should be considered: First, top–down selective attention to relevant information (Fritz et al., 2007) could facilitate target processing by enhancing the neural response to the attended stream (i.e., gain control; Lee et al., 2013). Second, top–down selective inhibition of maskers (Melara et al., 2002) could help to direct limited processing capacities away from irrelevant information (Desimone and Duncan, 1995), thereby avoiding full processing of distractors (Foxe and Snyder, 2011).

In this regard, interference of auditory maskers might be the result of both insufficient attention to the target and poor inhibition of noise and distractors. In this perspective article we focus on the latter, that is, neural mechanisms of auditory selective inhibition. We propose that cortical alpha (~10 Hz) oscillations are an important tool for top–down control as they regulate the inhibition of masker information during speech processing in challenging listening situations.

## 2. The functional significance of alpha oscillations

Neural oscillations in the alpha frequency range (~10 Hz) are the most dominant signal measurable in the human magneto- and electroencephalogram (M/EEG), going back to their first description by Berger (1931). The earliest observations of the alpha rhythm revealed that its amplitude is enhanced in humans who are awake but not actively engaged in any task. This finding led initially to the view that high alpha power might simply reflect the default state of brain inactivity or “cortical idling” (for a review, see Pfurtscheller et al., 1996).

Only within the last two decades, the functional significance of alpha oscillations has been recognized and furthermore its ubiquitous role across sensory modalities (visual: for review see Mathewson et al., 2011; sensorimotor: e.g., Haegens et al., 2012; auditory: e.g., Hartmann et al., 2012) and cognitive tasks (working memory: e.g., Jensen et al., 2002; attention: for a review see Klimesch, 2012; decision making: e.g., Cohen et al., 2009). One unifying mechanism suggested for alpha rhythms across modalities and brain areas is that it provides a neural means to functionally inhibit the processing of currently task-irrelevant or task-detrimental information (Jensen and Mazaheri, 2010; Foxe and Snyder, 2011). Please note that the opposite mechanism also has been proposed where higher inter-areal alpha phase synchronization does not index cortical inhibition but increased information processing such as for internal (working memory related) information processes (Palva and Palva, 2011). The functional inhibition hypothesis, though, has received neurophysiological support. For example, both alpha power (i.e., squared amplitude) and alpha phase modulate neuronal spike rate (Haegens et al., 2011) and thus can directly affect the efficiency of neural information flow. In future, the alpha network needs to be further characterized by its phase–amplitude coupling to gamma oscillations (Jensen et al., 2012) and its role in top–down control as implemented in different cortical layers (Buffalo et al., 2011; Spaak et al., 2012) or in thalamico-cortical communication (Strauss et al., 2010; Roux et al., 2013).

Despite the abundance of studies on the role of alpha activity for visual selective inhibition, there are currently few studies that directly examine the role of alpha activity in the auditory modality. Recently, a series of studies found modulations in alpha power in a variety of auditory tasks prompted by degraded spectral detail (Obleser and Weisz, 2012), missing temporal expectations (Wilsch et al., 2014), working memory load (Leiberg et al., 2006; Obleser et al., 2012), or syntactic complexity (Meyer et al., 2013). Together, these findings provide good evidence that alpha oscillatory power can be a reliable indicator of auditory cognitive load (see also Luo et al., 2005; Kaiser et al., 2007). In the following section, we argue that part of this cognitive load occurs due to auditory selective inhibition as a compensatory mechanism for demanding listening situations and manifests in enhanced alpha power.

## 3. Alpha oscillations as a tool for auditory selective inhibition

**Figure 1:**
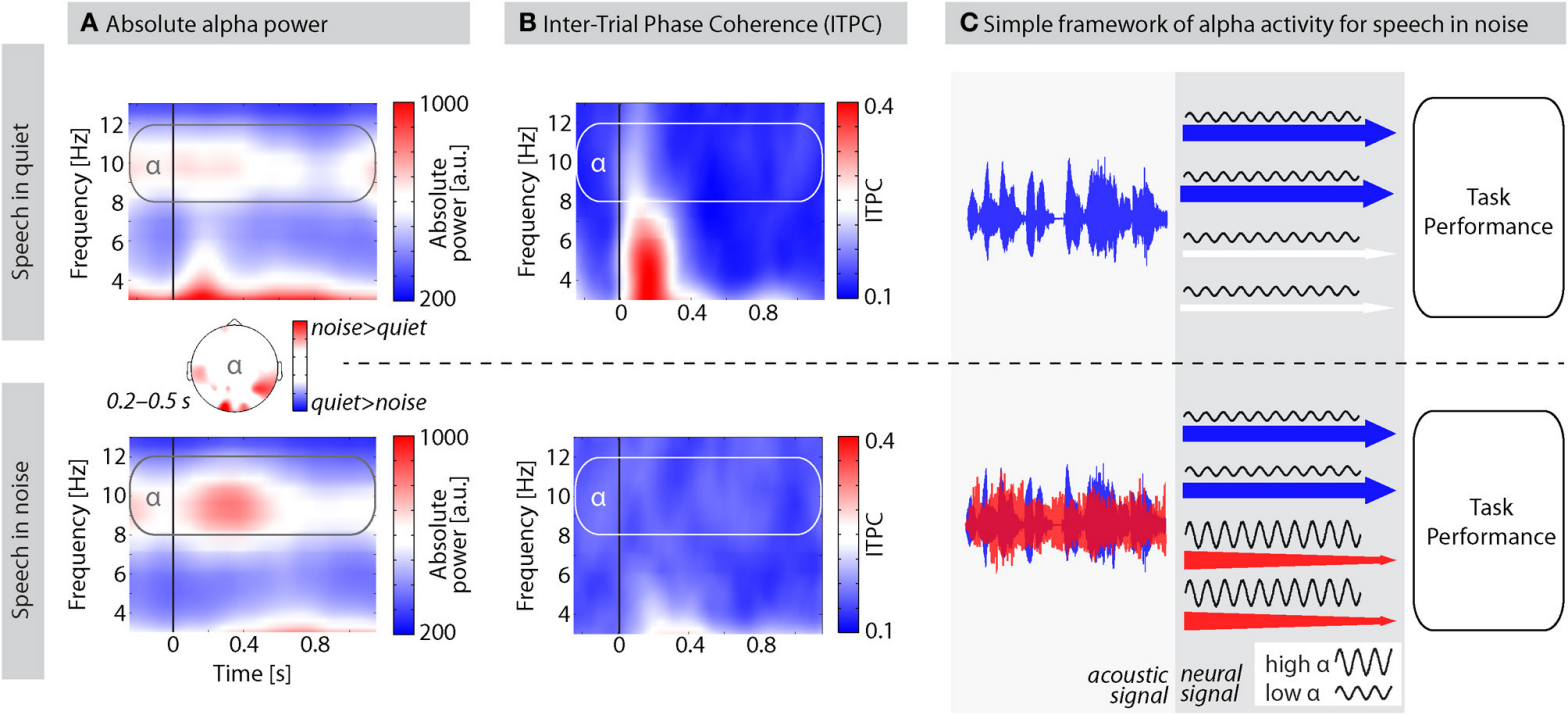
**The proposed role of alpha activity for speech processing in noise. (A)** Average absolute alpha power of 11 participants performing a lexical decision task on words in quiet (top) and in white noise (bottom). SNRs were titrated individually using a two-down-one-up staircase adaptive tracking procedure. Average SNR was −10.22 dB ±1.95 (*SD*) such that participants performed about 71% correct. Speech onset is indicated by the black vertical line at 0 s; average word length = 750 ms; EEG recorded from 61 scalp electrodes; time-frequency analysis using Morlet wavelets. Plots show measures of absolute power averaged over all scalp electrodes. Topography depicts the alpha power difference for speech in noise–quiet. Data were SCD (source current density)-transformed before power estimation to improve spatial resolution. **(B)** Inter-trial phase coherence (ITPC) as a measure of phase-locking of oscillations over trials. ITPC is bound between 0 and 1; higher ITPC values indicate stronger phase alignment across trials. **(C)** A simple framework of alpha oscillations for speech processing in noise. Acoustic signals overlap energetically as they enter the ear. At the brain level, features of speech and noise are processed as far as possible in distinct processing channels (depicted here with arrows; for details see text). High alpha power inhibits channels processing noise features to allow for an optimal task performance with minimized noise interference.

A common observation from our laboratory is a prominent increase in alpha power when participants listen to auditory materials presented against background noise (e.g., Wilsch et al., 2014). Figure 1A, for example, shows the grand average alpha power of 11 participants during a lexical decision task on isolated words presented in quiet (published in Strauß et al., 2014) and in white noise. For words in quiet, alpha power at around 10 Hz did not considerably increase after word onset. However, when words were presented in noise, alpha power was increased during the first 500 ms after word onset corresponding to the first two thirds of the average word duration. This effect was strongest over temporal and occipital sites (topography in Figure 1A) suggesting the inhibition of the task irrelevant visual modality but also compensatory mechanisms within speech-related areas. Critically, alpha power difference did not depend on ITPC (inter-trial phase coherence) differences, as indicated by the absence of a stronger ITPC in noise compared to quiet (Figure 1B). In fact, no significant ITPC differences were observed between 0.2 and 0.5 s. We therefore presume that induced (i.e., not strictly stimulus-locked; Freunberger et al., 2009) alpha power is crucial for speech processing in challenging listening conditions as it suppresses irrelevant information.

Figure 1C illustrates a tentative framework for how alpha oscillations could support auditory selective inhibition. Sounds arriving at the listener's ear must be further processed in the brain to extract task-relevant information. One way to think about the proposed mechanism is in terms of auditory object selection which requires object formation in the first place (Shinn-Cunningham, 2008). An auditory object might be formed on the basis of common spectro-temporal features, harmonicity, simultaneous onsets, or spatial grouping (Griffiths and Warren, 2004; Bizley and Cohen, 2013). We refer to all these different features used to form auditory objects as “channels” of auditory information represented by the arrows in Figure 1C. The concept of channels has a long tradition (Broadbent, 1958) and is inspired by the most clear distinction of target and distractor used in many dichotic listening paradigms where left and right ear channel need to be separated. Nevertheless, channels in our framework should be conceived as functional auditory processing units rather than anatomical pathways. As soon as these channels are defined, attention or inhibition can be selectively applied, given attentionally flexible fields in the auditory cortices (Petkov et al., 2004). Note that even though in the visual modality claims about alpha oscillations in feature-based (Romei et al., 2012) and object-based (Kinsey et al., 2011) attention have been made, we do not make any assumption about this distinction in our framework and use the term “channels” for both features and objects, or early and late selection.

If speech is presented in quiet (Figure 1C, top panel), alpha power is low in channels processing features of the speech signal to support processing of task-relevant information. Accordingly, the net resulting alpha power in the M/EEG would continue on baseline level (Figure 1A) and decrease during word integration (>400 ms). If, however, speech is presented in the presence of maskers (e.g., environmental noise, distracting talkers; Figure 1C, bottom panel), alpha power needs to be up-regulated first in those channels processing noise features before it is going to be suppressed during word integration (Figure 1A). Enhanced alpha activity inhibits processing of noise and thereby “protects” (Klimesch, 1999; Roux and Uhlhaas, 2014) the task- or performance-relevant information in the speech signal from noise interference.

Importantly, the up-regulation of alpha power in channels that process noise is not an automatic (“bottom–up”) process but critically depends on “top–down” attentional control. For instance, in a multi-talker situation, target and distracting talker switch roles permanently, as the listener decides to change the conversational partner. In such a situation, M/EEG alpha power would be constantly at a high level; however, the deployment of alpha power onto the different processing channels would be changing continuously.

What is the functional role of high alpha activity for word processing in noise? To answer this question, it is essential to distinguish between interpretations in which alpha activity is related to target processing from these related to noise processing. It is possible that the reduced intelligibility of words in noise leads to sub-optimal word processing and thus to less alpha suppression in brain areas relevant for speech processing (Strauß et al., 2014). The inverse mechanism, as we put forward in the current framework, is equally likely by which alpha power is enhanced for temporarily irrelevant information and thereby compensates for perceived cognitive effort (increased when listening to speech in noise: Larsby et al., 2005; Helfer et al., 2010; Zekveld et al., 2011). In this regard, alpha would “protect” the lexical processes from noise interference. The challenge will be to experimentally dissect these (not mutually exclusive) mechanisms. We now review initial evidence for alpha's inhibitory role in audition.

Currently, there are only few studies that show alpha power modulations when participants simultaneously listen to two auditory streams, that is, one signal and one masker. In one study by Kerlin et al. (2010), participants were simultaneously listening to two spatially separated speech streams. On each trial, an initial visual cue indicated whether they were supposed to attend the left or right stream. During speech presentation, EEG alpha power was enhanced over the cerebral hemisphere contralateral to the masker, while alpha power was reduced contralateral to the to-be-attended stream. The authors concluded that this alpha lateralization indexes the direction of auditory attention to speech in space. Importantly, this finding corroborates our view that enhanced alpha power in brain areas engaged in distractor processing decreases further processing of the distractor and hence, facilitates processing of the target signal. However, two questions arise from this study: First, as the direction of auditory attention was cued visually in this study, it might be that the alpha lateralization indicates the allocation of supramodal rather than auditory selective attention (Farah et al., 1989). Second, spatial attention may play a special role not least because of auditory processing models suggesting separate what- and where-pathways (Rauschecker and Scott, 2009).

In three other recent studies, alpha power modulations were consistently found during the anticipation of auditory target signals from the left or right (Banerjee et al., 2011; Müller and Weisz, 2012; Ahveninen et al., 2013). In these studies, participants were cued to attend either the auditory event on the left or right, and to ignore the distractor on the other side. Alpha power was enhanced during the anticipation of auditory stimulation contralateral to the distractor. These results demonstrate alpha lateralization effects already during the preparation for an auditory selective listening task. This is in line with studies reporting high pre-stimulus alpha power when participants are about to miss a (visual) target (van Dijk et al., 2008; Busch et al., 2009; Romei et al., 2010). In terms of our framework (Figure 1C), anticipatory high alpha power successfully blocks in-depth processing of sensory information that might lead to missing the target.

However, interpretations of these studies are limited for our model, since alpha power modulations were found only during the anticipation but not during the actual processing of competing auditory streams. More data are clearly needed on the peri-stimulus alpha dynamics. As the spatial resolution of M/EEG is limited, prospective experiments could induce alpha oscillations over specific brain areas using transcranial alternating current stimulation (tACS) to assess the influence of alpha modulations on listening success under adverse acoustic conditions. Moreover, future studies could record the electrocorticogram (ECoG) directly from the cortical surface to track alpha sources and reveal the interplay between frequency bands. Such higher spatial resolution would allow to differentiate between alpha activity in brain regions associated with processing the masker or the signal. As of now, we are left to speculate how spatially specific alpha oscillations might operate, for example along a cochleotopic gradient in primary auditory cortex. The best data to infer from stems from visual cortex, where for example Buffalo and colleagues recorded with two electrode tips in attended vs. non-attended receptive fields less than a millimeter apart and report attention-dependent opposing, and deep-layer-specific alpha changes (expressed as alpha spike-field coherence; Buffalo et al., 2011). Comparable data are, to our knowledge, still missing for auditory areas.

In the next two sections, we will elaborate first, at which levels of auditory processing alpha power might be deployed for the inhibition of different kinds of auditory maskers, and second, how age and hearing loss might affect auditory selective inhibition.

## 4. Masking release via alpha enhancement along the auditory pathway

So far, we have shown that alpha oscillations are an attractive neural candidate mechanism of selective auditory inhibition. There are different aspects which need to be systematically investigated in order to determine the role of alpha: Which neural circuits “deploy” or trigger high-alpha states? And in terms of the current framework: What kind of channels can be attenuated by enhanced alpha power?

Currently, there are few studies mapping the sources of alpha power during masked auditory processing. Some evidence has accumulated showing noise-invariant representations of the signal in auditory cortices (Chang et al., 2010; Ding and Simon, 2012) with the degree of invariance increasing from peripheral to cortical processing stages (Rabinowitz et al., 2013). If we assume that alpha is an important central mechanism to inhibit various types of maskers, these studies suggest that masking release via alpha enhancement might occur as early as in primary auditory cortex. A first direct hint to this idea might be the case of an illusory sound percept like tinnitus, which can be centrally suppressed by means of increasing alpha power in primary auditory cortex (Leske et al., 2013; Weisz et al., 2014). This is in line with research showing that attention modulates activity in sensory cortices corresponding to the modality of the stimulus (e.g., Heinrich et al., 2011; Wild et al., 2012). Thus, alpha activity in primary auditory cortex might be crucially contributing to inhibiting the formation of auditory objects.

In future studies investigating underlying alpha sources, a distinction between energetic and informational masking might be crucial (Brungart et al., 2001; Mattys et al., 2009; Scott and McGettigan, 2013; for a more comprehensive overview of potential adverse listening conditions see Mattys et al., 2012). Energetic masking describes the competition of auditory target and masker in the auditory periphery due to spectro-temporal overlay of the two signals, causing an overlap of excitation patterns in the cochlea and auditory nerve (Durlach et al., 2003). One type of background signal often assumed to cause primarily energetic masking is white noise (e.g., Arbogast et al., 2005) which is quasi-stationary and has high energy in a broad frequency range (for discussion see Stone et al., 2012). Although informational masking is sometimes defined only negatively as all masking effects not accounted for by energetic masking (cf. Gutschalk et al., 2008), a more refined definition is required, especially when it comes to speech processing. When target speech is masked by a competing talker, it is not just the energetic overlap of the two signals that causes masker interference. Rather, the speech masker initiates phonetic and semantic processing that interferes with the linguistic processing of the target (Schneider et al., 2007). Thus, informational masking describes the interference of target and masker at a more central, cognitive level, whereas energetic masking refers to energetic overlap in the auditory periphery.

According to the framework described above, alpha oscillations might be important for inhibition of both types of maskers, however, in different brain areas. We presume that energetic maskers are inhibited by enhanced alpha activity in auditory cortex (Müller and Weisz, 2012). In contrast, processing of informational maskers like competing speech should rather be inhibited by alpha activity in higher auditory areas such as posterior superior temporal gyrus (pSTG) and beyond, relevant for linguistic processing (Scott et al., 2004, 2009). In addition to the proposed inhibition of auditory input, alpha oscillations are involved in supramodal or crossmodal inhibition of the currently task-irrelevant modality (Banerjee et al., 2011).

## 5. Effects of age and hearing loss on auditory distractor inhibition

In acoustically demanding multi-talker situations, older listeners typically experience more difficulties compared with younger adults. It is however unclear, in how far these difficulties are caused by age-related decline in perceptual auditory acuity (hearing loss or loss of temporal and spectral resolution; Fostick and Babkoff, 2013), decline of cognitive functioning with age, or both (Wingfield et al., 2005). Crucial for the present framework, however, both auditory perceptual and cognitive decline could lead to insufficient masker inhibition. First, compared with normal-hearing controls, listeners with hearing loss are less successful in utilizing spectral (Lorenzi et al., 2006), temporal (Tremblay et al., 2003), and spatial auditory cues (Neher et al., 2009) important for the perceptual segregation of different sound sources. Thus, attending to relevant and inhibiting irrelevant sound sources is impaired, as auditory features are lacking to distinguish the different sound sources in the first place (Shinn-Cunningham and Best, 2008). Second, age negatively affects many aspects of cognitive functioning (Park et al., 2003), amongst it the ability to suppress irrelevant but salient auditory distractors (Chao and Knight, 1997; Tun et al., 2002; Passow et al., 2014). Thus, even if the perceptual segregation of sound sources is accomplished successfully, the insufficient inhibition of maskers may cause interference.

In line with prior studies that found age effects on brain oscillatory activity in the alpha frequency range (Yordanova et al., 1998; Klimesch, 1999; Böttger et al., 2002), we consider it valuable to investigate alpha oscillations in demanding listening tasks as an indicator of age-dependent auditory cognitive effort of masker inhibition. We presume that auditory selective inhibition, realized by alpha activity in channels relevant for masker processing (Figure 1C), serves as a compensatory mechanism as multi-talker listening conditions become more demanding, for instance due to a decreasing signal-to-noise ratio (SNR). The study of alpha oscillations could help to reveal how listeners of different age exert top–down attentional control to facilitate processing of task-relevant signals and inhibit processing of interfering maskers. In particular, this line of research might foster the understanding of why older listeners find it more exhausting to participate in cocktail party-like listening situations compared with younger listeners (Pichora–Fuller, 2003).

## 6. Conclusions

In this perspective article, we have presented a framework for studying alpha oscillations as a tool for auditory selective inhibition in challenging listening situations. We have presented initial evidence qualifying alpha oscillations as a pivotal mechanism affecting listening in multi-talker situations. Future studies could expand these findings and study the role of alpha oscillations (1) during speech perception in ecologically valid listening situations, (2) in the presence of energetic and informational maskers, and (3) for aging and hearing-impaired listeners.

### Conflict of interest statement

The authors declare that the research was conducted in the absence of any commercial or financial relationships that could be construed as a potential conflict of interest.
